# An Exploratory Study of the Association between *KCNB1* rs1051295 and Type 2 Diabetes and Its Related Traits in Chinese Han Population

**DOI:** 10.1371/journal.pone.0056365

**Published:** 2013-02-19

**Authors:** Yu-Xiang Zhang, Yan Liu, Jing Dong, You-Xin Wang, Jing Wang, Guo-Qing Zhuang, Shu-Jing Han, Qing-Qing Guo, Yan-Xia Luo, Jie Zhang, Xiao-Xia Peng, Ling Zhang, Yu-Xiang Yan, Xing-hua Yang, Hong Wang, Xu Han, Guang-Xu Liu, You-Hou Kang, You-Qin Liu, Sheng-Feng Weng, Hong Zhang, Xiao-Qiang Zhang, Ke-Bao Jia, Li Wang, Lei Zhao, Zhong-Xin Xiao, Shu-Hua Zhang, Hui-Hui Wu, Qing-Xuan Lai, Na Qi, Wei Wang, Herbert Gaisano, Fen Liu, Yan He

**Affiliations:** 1 Department of Epidemiology and Health Statistics, School of Public Health and Family Medicine, Capital Medical University, Beijing, China; 2 Beijing Municipal Key Laboratory of Clinical Epidemiology, Beijing, China; 3 Infection Control Office, Peking University People’s Hospital, Beijing, China; 4 Health Medical Center, Beijing Xuanwu Hospital, Capital Medical University, Beijing, China; 5 Department of Endocrinology, Beijing Xuanwu Hospital, Capital Medical University, Beijing, China; 6 College of Life Science, Graduate University of Chinese Academy of Sciences, Beijing, China; 7 Departments of Medicine and Physiology, University of Toronto, Toronto, Ontario, Canada; 8 Department of Clinical Laboratory, Beijing Geriatric Hospital, Beijing, China; 9 Experimental Teaching Center, Capital Medical University, Beijing, China; 10 Center for Clinical Laboratory, Capital Medical University, Beijing, China; Baylor College of Medicine, United States of America

## Abstract

Since the KCNB1 encoding Kv2.1 channel accounts for the majority of Kv currents modulating insulin secretion by pancreatic islet beta-cells, we postulated that KCNB1 is a plausible candidate gene for genetic variation contributing to the variable compensatory secretory function of beta-cells in type-2 diabetes (T2D). We conducted two studies, a case-control study and a cross-section study, to investigate the association of common single-nucleotide polymorphisms (SNPs) in KCNB1 with T2D and its linking traits. In the case-control study, we first examined the association of 20 tag SNPs of KCNB1 with T2D in a population with 226 T2D patients and non-diabetic subjects (screening study). We then identified the association in an enlarged population of 412 T2D patients and non-diabetic subjects (replication study). In the cross-sectional study, we investigated the linkage between the candidate SNP rs1051295 and T2D by comparing beta-cell function and insulin sensitivity among rs1051295 genotypes in a general population of 1051 subjects at fasting and after glucose loading (oral glucose tolerance tests, OGTT) in 84 fasting glucose impaired subjects, and several T2D-related traits. We found that among the 19 available tag SNPs, only the KCNB1 rs1051295 was associated with T2D (*P* = 0.027), with the rs1051295 TT genotype associated with an increased risk of T2D compared with genotypes CC (*P* = 0.009). At fasting, rs1051295 genotype TT was associated with a 9.8% reduction in insulin sensitivity compared to CC (*P* = 0.008); along with increased plasma triglycerides (TG) levels (TT/CC: *P* = 0.046) and increased waist/hip (W/H) ratio (TT/CC: *P* = 0.013; TT/TC: *P* = 0.002). OGTT confirmed that genotype TT exhibited reduced insulin sensitivity by 16.3% (*P* = 0.030) compared with genotype TC+CC in a fasting glucose impaired population. The KCNB1 rs1051295 genotype TT in the Chinese Han population is associated with decreased insulin sensitivity and increased plasma TG and W/H ratio, which together contribute to an increased risk for T2D.

## Introduction

Although the precise mechanisms underlying the development and progression of T2D remain unclear, a consensus is that pathophysiologic defects underlying T2D include insulin resistance of peripheral tissues and defects in pancreatic islet insulin secretory capacity [1], each influenced by environmental and genetic factors [2]. With respect to the latter, numerous genome-wide association studies (GWAS) have been conducted leading to identification of susceptibility loci for T2D. SNPs of ion channel genes contributing to beta-cell secretory defects in T2D include ATP-sensitive K^+^ (K_ATP_) channels (KCNJ11) [3] and Ca^2+^ channels (CACNA1E) [4]. K_ATP_ channel closure causes cell depolarization which opens Ca^2+^ channels to enable Ca^2+^ influx that evokes insulin exocytosis, and thus defects in these ion channels would result in insulin secretory insufficiency [5]. Most recently, studies revealed SNPs in KCNQ1, encoding Kv7.1, associated with T2D in the Japanese population [6], and confirmed to be present in Chinese [7], [8], Koreans [6], and also Swedes [6] and Danes [9]. Furthermore, common variants of KCNQ1 have been shown to be associated with reduced insulin granule docking and depolarization-evoked insulin exocytosis [10], and impairment in insulin secretion during glucose loading [11].

Voltage-gated K^+^ (Kv) channels regulate cell membrane repolarisation that controls duration of Ca^2+^ channel opening [12], which in beta-cells influences duration of insulin secretion [13], [14]. Kv7.1 is however not the major Kv channel in pancreatic islets, but rather in the heart, where genetic defects account for several cardiac arrhythmic disorders [15], [16]. The dominant Kv channel accounting for ∼70% of membrane repolarisation in beta-cells is Kv2.1 [13], [14], encoded by KCNB1 located in chromosome 20q13.2 [17]. The Kv2.1 channel is also expressed in brain, atria, ventricle, skeletal muscle and other tissues [17]. Based on our previous work on the beta-cell Kv2.1 channel identifying its important role in modulating of insulin secretion [18], [19], we pursued the possibility and hypothesis that there may be a genetic variation in KCNB1 associated with T2D that could influence disease progression and/or compensatory capacity. This prompted us to search for candidate KCNB1 SNPs associated with T2D, employing a case-control study, followed by a cross-section study to examine underlying type 2 diabetic-related traits linking this association in the Chinese Han population.

## Materials and Methods

This study was approved by the Ethics Committees of Capital Medical University (Beijing, China) and also the Beijing Geriatric Hospital and Beijing Xuanwu Hospital, and was conducted in accordance with the principles of the Helsinki Declaration II. Written consent was obtained from all participants.

### Study Participants and Study Design

In the case–control study, the participants were composed of 412 Chinese Han participants. This included 176 unrelated individuals with T2D designated as Cases, identified by 1999 WHO criteria [20]. These subjects were recruited consecutively from the Department of Endocrinology of two Beijing hospitals, Xuanwu Hospital Capital Medical University and Beijing Geriatric Hospital. 236 unrelated non-diabetic individuals were recruited consecutively, designated as Controls, from the Departments of Otolaryngology and Ophthalmology of the above two hospitals during the same period as cases. These controls were identified by having a fasting plasma glucose level of <6.1 mmol/L and having not been diagnosed as T1D or T2D.

For the first stage of the case-control study, a KCNB1 gene-wide tag SNPs-based association study (screening study) was performed, aimed at screening for association between the presence of KCNB1 SNPs and T2D. 226 participants were randomly selected from the above 412 participants, which included 112 individuals with T2D as cases and 114 non-diabetic individuals as controls**.** In the initial screening study, the KCNB1 3′-UTR rs1051295 (T>C) was found to have a trend towards association with T2D, which prompted us to perform a replication study aimed at identifying a genuine association of the KCNB1 rs1051295 and T2D. The participants included all 412 Chinese Han participants.

Then in the cross-section study, we examined the underlying mechanism linking the association of KCNB1 rs1051295 and T2D. 1051 individuals participating in annual health examinations were consecutively recruited at the Physical Examination Center, Beijing Xuanwu Hospital. Here we tested for the association of some T2D-related traits with KCNB1 rs1051295 at basal condition. Individuals with previously diagnosed T1D or T2D or with fasting plasma glucose of ≥6.1 mmol/L were excluded.

We also investigated whether KCNB1 rs1051295 is associated with two major pathogenic contributing factors to T2D: beta-cell compensatory secretory function and peripheral insulin sensitivity at glucose load. 84 volunteer outpatients with fasting glucose between 6.1 and 7.0 mmol/L who willing to undertake an OGTT, were recruited consecutively from the Department of Endocrinology of the Beijing Xuanwu Hospital. For the OGTT study, participants drank a 75 g glucose solution, and blood samples were drawn at 0, 30, 60 and 120 minutes and sent for glucose and insulin determination, and genotyping.

From all participants, blood samples were drawn for DNA preparation and determination of fasting plasma glucose, fasting insulin, triglycerides (TG), total cholesterol (TCH), high-density lipoprotein cholesterol (HDL-C), low-density lipoprotein cholesterol (LDL-C). A physical examination was performed on all participants to obtain their height, weight, waist circumference, hip circumference, and blood pressure.

### Genotyping, PCR and Quality Control

We used genotyping data from the CHB panel (Han Chinese in Beijing) of the phase II HapMap Project to select 20 tag SNPs within the range of 5000 bp upstream of the initiation codon and 5000 bp downstream of the termination codon of KCNB1 on the basis of the following principal criteria: r^2^>0.8 and minor allele frequency (MAF)>0.1. Of those tag SNPs, 19 SNPs were located in introns, and 1 was in the 3′-UTR. Detailed information for all the tag SNPs (including genomic position, genic position, minor allele frequency, Hardy-Weinberg equilibrium) are shown in Table1.

**Table 1:** KCNB1 gene tag SNPs information.

SNP ID	Genomic position (bp)	Genic position	Alleles (major/minor)	MAF	HWE (*P*)
rs1051295	47988905	3′-UTR	T/C	0.45	1.00
rs186942	48045742	Intron	G/C	0.24	0.85
rs1961192	48006307	intron	T/C	0.47	0.41
rs237451	48024332	intron	G/A	0.33	0.46
rs237458	48031549	intron	C/T	0.44	1.00
rs237476	48051382	intron	G/T	0.24	0.17
rs237477	48057448	intron	T/C	0.28	0.34
rs3787318	48058067	intron	T/C	0.10	1.00
rs4810952	48006175	intron	T/C	0.38	0.90
rs553213	48097564	intron	T/C	0.35	1.00
rs562954	48092076	intron	G/A	0.20	1.00
rs572845	48076614	intron	C/T	0.11	0.74
rs579113	48075912	intron	A/G	0.23	0.27
rs610412	48078202	intron	A/C	0.16	0.46
rs6125647	48027463	intron	A/C	0.24	0.91
rs653070	48087408	intron	C/T	0.31	0.33
rs7269864	48096608	intron	T/C	0.11	0.87
rs742759	48061145	intron	G/A	0.13	1.00
rs802952	48086269	Intron	T/C	0.22	0.19
rs926673	48030010	intron	T/G	0.19	0.05

MAF: minor allele frequency. HWE: Hardy-Weinberg equilibrium.

Genomic DNA was extracted from peripheral white blood cells of participants using a DNeasy tissue kit (Qiagen) according to the manufacturer’s instructions. All SNPs were genotyped using MassArray (Sequenom, San Diego, CA) based on allele-specific MALDI-TOF mass spectrometry. DNA from cases and controls was randomly assigned to 96 well plates, and blinded genotyping was performed. The call rates for the genotyping of the SNPs were all >90% except for rs802952, for which the rate was 39.58%. Thus rs802952 was excluded from further analysis.

For the replication study and the cross section study, rs1051295 was genotyped using PCR-based pyro-sequencing technology and DNA sequencing with an ABI 3730 automated sequencer (Applied Biosystems, Foster city, CA). The primers are F: biotin-GCGCAAAACCCTTACTCA AAT and R: GCCAGGGGGCAATTAGAAT for PCR amplification and 5′- TGGTATCTCAA AATAAAATC-3′ for sequencing. The primers were designed according to the published sequence of KCNB1 (http://www.ncbi.nlm.nih.gov/pubmed/), the GenBank accession number is NT_002370.

PCR was conducted in a 50-µl reaction mixture containing 100 ng genomic DNA, 0.5 pmol primer, 2× master mix (mixture of reaction buffer, MgCl_2_ and DNA polymerase) (ToYoBo, Japan). PCR products were denatured for 2 min at 95°C and then thermal-cycled for 30 s at 95°C, 30 s at 59°C, and 60 s at 72°C, repeating the cycle 40 times. A final extension step at 72°C for 10 min completed the program. Pyrosequencing analysis was performed on Streptavidin Sepharose™ HP (Amersham, Sweden). ssDNA prepared from 50 ul of biotinylated PCR product was then subjected to pyrosequencing. The call rates was 96.02% (169/176) and 94.92% (224/236) for cases and control, respectively, for the replication study, and 97.91% (1029/1051) and 100% (84/84), respectively, for the first and second stages of the cross-section study.

For both the case-control and the cross-section studies, genotyping was repeated in 5% of random samples for verification and quality control, which all revealed the genotype data had an error rate of <1%.

### Assessment of Islet Beta-cell Secretory Function and Insulin Sensitivity

We employed the homeostatic model to calculate beta-cell function (HOMA-B%) and insulin sensitivity (HOMA-S%) at basal condition (the software HOMA calculator downloaded from www.dtu.ox.ac.uk/homa). In the glucose loading condition (OGTT), insulin sensitivity was assessed by the Matsuda index = 10,000/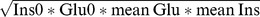. Ins0 is fasting insulin, Glu0 is fasting glucose. The islet beta-cell secretory function was assessed by the Insulin Secretion-Sensitivity Index-2 (ISSI-2) = AUC (insulin curve)/AUC (glucose curve)* Matsuda index. The Matsuda index is a validated OGTT-based measure of insulin sensitivity that is analogous to the disposition index obtained from iv glucose tolerance tests [21]. Area under curve (AUC) for glucose and insulin were calculated by the trapezoidal rule.

### Statistical Analyses

In the case-control study, the χ^2^ test was used to examine the differences in gender between case and control groups. The differences between T2D-related traits in T2D case and control groups were compared by an independent–sample *t* test. The distribution of SNPs in Cases and Controls were compared using the χ^2^ test and an unconditional Logistic regression analysis, in which the association of a SNP with T2D was adjusted for age, gender and BMI. T2D-related traits among the three genotypes of rs1051295 were compared by one-way ANOVA test.

The program Haploview (http://www.broad.mit.edu/mpg/haploview/) was used to calculate pair-wise linkage disequilibrium statistics and to test allelic and haplotype associations with T2D.

In the cross-section study, we used independent-samples *t* test to compare T2D-related traits between genotypes (TT vs TC+CC) in OGTT, or one-way ANOVA test among 3 genotypes at fasting. Association of rs1051295 genotypes with HOMA-B%, HOMA-S%, ISSI-2, Matsuda index and other T2D-related traits were examined by multiple linear regression analysis, in which association was adjusted for age, gender, and BMI.

Hardy–Weinberg equilibrium was determined for each SNP distribution. All analysis was done on SPSS software, version 18.0 (purchased by Capital Medical University, China). All tests were two-tailed, with a significance level of 0.05. Data were expressed as means ± SD, unless the data did not conform to a normal distribution, in which case the data were expressed as median and quartiles and natural Log-transformed for analysis.

## Results

### Candidate-gene Association Study Identified KCNB1 3′-UTR rs1051295 is Likely to be Associated with T2D

The demographic and clinical characteristics of the participants for the case-control study are summarized in Table 2. Among the 19 available tag SNPs, the distribution of rs742759 (*P* = 0.16) and rs1051295 (*P* = 0.18) showed a possible association with T2D. The other 17 tag SNPs did not show a trend of association with T2D (*P*>0.20, data not shown). However, only rs1051295 had a trend towards association with T2D (*P* = 0.08) when adjusted for age, gender and BMI (Table 3).

**Table 2:** Characteristics of the participants in the case-control study.

Variables	Screen study			Replication study		
	Case (n = 112)	Control (n = 114)	*P*	Case (n = 176)	Control (n = 236)	*P*
Age (years)	66.96±13.32	67.32±15.25	0.85	65.10±14.06	63.92±15.01	0.42
M/F	56/56	68/46	0.15	88/88	124/112	0.61
BMI (kg/m^2^)	25.21±4.33	23.69±4.20	**0.01**	25.31±4.29	23.53±3.99	**<0.001**
W/H ratio	0.90±0.07	0.89±0.08	0.37	0.89±0.07	0.88±0.09	0.27
TG (mmol/L)	2.15±1.78	1.27±0.62	**<0.001**	2.02±1.57	1.41±0.75	**<0.001**
SBP(mmHg)	129.74±16.23	126.35±17.99	0.11	129.91±15.61	126.37±17.73	**0.04**
DBP (mmHg)	74.55±9.41	76.06±12.00	0.30	75.87±9.50	76.05±12.00	0.87
FPG (mmol/L)	9.29±3.06	5.29±0.75	**<0.001**	9.16±3.68	5.50±0.79	**<0.001**

M/F: male/female. W/H ratio: waist/hip circumference. TG: triglycerides. SBP: systolic blood pressure. DBP: diastolic blood pressure. FPG: Fasting plasma glucose. Data are presented as mean ±SD. *P* from independent-sample *t* test except *P* for M/F from χ^2^ test.

**Table 3:** Distribution of two tag SNPs of KCNB1 in the screening study.

SNP ID	Cases	Controls	χ^2^	*P^1^*	*P^2^*	OR (95% CI)
rs1051295	n = 110	n = 111	3.41	0.18		
CC	13	23				
TC	62	59				
TT	35	29				
TT vs CC					0.08	2.18(0.89–5.31)
TT vs TC					0.15	1.81(0.89–4.09)
rs742759	n = 103	n = 110	4.63	0.16		
GG	69	86				
GA	23	18				
AA	11	6				
AA vs GG					0.19	1.44(0.84–2.56)
AA vs GA					0.56	1.45(0.42–4.96)

*P^1^*: from χ^2^ test; *P^2^*: from Logistic regression analysis and adjusted for age, gender and BMI. OR: odds ratio. CI: confidence interval.

To determine whether these SNPs demonstrated any additional evidence of association with T2D when examined together, haplotypes for 19 SNPs were constructed using the Haploview program. We identified four haplotypes for the KCNB1 gene, and found no association between any of them and T2D (data not shown). It is noteworthy that rs1051295 was not in a haplotype block generated in this study due to weak linkage disequilibrium with other SNPs.

In order to identify this association, a replication study was performed in the source population of the above screening study. This population consisted of 176 cases and 236 controls. Hardy–Weinberg equilibrium testing showed *P* = 0.06 and 0.14 for rs1051295 genotypes in cases and controls, respectively. TT genotype of rs1051295 demonstrated a significantly increased risk for T2D compared with the CC (*P* = 0.009, OR = 2.58, 95% CI = (1.27, 5.23)), but not with TC (*P* = 0.226, OR = 1.36, 95% CI = (0.83, 2.25)) genotypes. In the dominant model, allele C (genotype CC and TC) did not show decreased risk for T2D compared with genotype TT (*P* = 0.071, OR = 0.64, 95% CI = (0.40, 1.04)); but in the recessive model, allele T(genotype TT+TC) showed increased risk for T2D compared with genotype CC(*P* = 0.02, OR = 2.10, 95% CI = 1.13,3.90) (Table 4).

**Table 4:** Distribution of KCNB1 rs1051295 SNPs in the replication study.

SNP ID	Cases	Controls	χ^2^	*P^1^*	*P^2^*	OR (95% CI)
rs1051295	n = 169	n = 224	7.21	0.027		
CC	21	48				
TC	93	123				
TT	55	53				
TT vs CC					**0.009**	2.58(1.27,5.23)
TT vs TC					0.226	1.36(0.83,2.25)
Dominentmodel				0.051	0.071	0.64(0.40,1.04)
Recessivemodel				**0.020**	**0.020**	2.10(1.13,3.90)

*P^1^*: from χ^2^ test; *P^2^:* from Logistic regression analysis and adjusted for age, gender and BMI. Dominant Model: CC+TC compared with TT; Recessive Model: TT+TC compared with CC. OR: odds ratio. CI: confidence interval.

We further compared a number of T2D-related traits among the three variants of rs1051295 including BMI, W/H ratio, TG, fasting plasma glucose, SBP, DBP as well as gender and age, but found no association of any genotype with these traits (Table S1 and Table S2).

### KCNB1 rs1051295 Associated with T2D-related Traits Under Basal Condition and OGTT

Since the pathophysiology underlying T2D is characterized by defective insulin secretion and reduced insulin sensitivity, a cross-section study was designed to determine beta-cell secretory function and insulin sensitivity in the three KCNB1 rs1051295 variants under basal (fasting) condition to assess which of these traits could account for their differential risk for T2D. Table 5 shows that the insulin sensitivity of genotype TT (90.1%) was reduced by 9.8% compared to genotype CC (99.9%), which was a significant difference (*P* = 0.008, b = −0.09, 95% CI (−0.16, −0.02)) after adjusting for age, gender and BMI. Comparing TT to TC (95.2%), the difference was 5.1%, which did not reach statistical significance (*P* = 0.09, b = −0.04, 95% CI (−0.09, 0.01)). When we combined genotype CC and TC as one group of allele C and compared its insulin sensitivity (HOMA-S%) with that of genotype TT in the dominant model, the result showed genotype TT decreased insulin sensitivity (*P* = 0.05, b = −5.22, 95% CI = (−10.49, −0.00)), whereas allele T (genotype TC and TT) did not show decreased insulin sensitivity compared with genotype CC in the recessive model (*P* = 0.08, b = −5.87, 95% CI = (−12.53,0.79)).

**Table 5:** Comparison of T2D-related traits among rs1051295 genotypes in a general population at fasting.

Variable	TT	TC	CC	TT vs TC			TT vs CC		
				*P^1^*	*P^2^*	b (95% CIs)	*P^1^*	*P^2^*	b (95% CIs)
N (%)	346 (33.62)	514(49.95)	169(16.42)						
M/F	153/193	226/288	74/95	0.94			0.93		
Age (years)	40.03±10.64	40.30±10.16	38.72±9.66	0.70			0.17		
BMI (kg/m^2^)	23.12±2.42	22.90±2.49	22.92±2.55	0.18	0.110	0.25 (−0.06,0.57)	0.38	0.565	0.12(0.30, 0.54)
W/H ratio	0.81±0.07	0.80±0.07	0.79±0.07	**0.02**	**0.002**	0.01(0.00,0.02)	**0.02**	**0.013**	0.01(0.00,0.02)
TG(mmol/L)*	1.04(0.74,1.46)	0.98(0.68,1.38)	0.93(0.67,1.31)	0.07	0.092	0.05(−0.01,0.11)	**0.02**	**0.042**	0.08(0.00, 0.16)
HDL-C(mmol/L)	1.65±0.34	1.65±0.35	1.67±0.29	0.91	0.873	−0.004(−0.05,0.04)	0.62	0.478	−0.02(−0.08, 0.04)
LDL-C(mmol/L)	2.72±0.78	2.64±0.66	2.63±0.68	0.14	0.337	0.05(−0.05, 0.15)	0.19	0.466	0.05(−0.08, 0.18)
TCH(mmol/L)	4.65±0.88	4.57±0.83	4.55±0.76	0.15	0.221	0.07(−0.04,0.18)	0.18	0.422	0.06(−0.09, 0.21)
FPG (mmol/L)	4.98±0.51	4.99±0.46	4.99±0.50	0.76	0.635	−0.02(−0.08,0.05)	0.73	0.362	−0.04(−0.12,0.04)
FINS(mUI/L)	9.08±3.51	8.60±3.26	8.14±3.07	**0.04**	0.091	0.38 (−0.06,0.81)	**0.00**	**0.004**	0.86(0.27, 1.44)
HOMA-S%*	90.12(68.55,116.15)	95.18(71.60,123.40)	99.85(78.40,126.98)	**0.04**	0.096	−0.04(−0.09,0.01)	**0.00**	**0.008**	−0.09(−0.16, −0.02)
HOMA-B%*	105.30(86.85,125.75)	102.30(80.90,121.00)	99.15(79.40,118.50)	**0.04**	0.068	0.04(−0.00, 0.08)	**0.01**	**0.004**	0.08(0.03, 0.13)

M/F: male/female. W/H ratio: Waist/hip circumference. TG: Triglycerides. HDL-C: high-density lipoprotein cholesterol. LDL-C: low-density lipoprotein cholesterol. TCH: total cholesterol. FPG: Fasting plasma glucose. FINS: Fasting insulin b: unstandardized coefficients, CI: confidence interval. *P^1^*: from one-way ANOVA test except for *P* for M/F from χ^2^ test; *P^2^,* b and 95% CIs*:* from multiple linear regression analysis adjusted for age, gender and BMI. *presented as median and quartiles and natural Log-transferred for analysis.

Importantly, the rs1051295 genotype TT was associated with an unfavorable W/H ratio and higher plasma TG levels compared to genotype CC (W/H ratio: *P* = 0.01, b = 0.01, 95% CI (0.00,0.02); TG: *P* = 0.04, b = 0.08, 95% CI (0.00, 0.16)) and TC (W/H ratio: *P* = 0.002, b = 0.01, 95% CI (0.00, 0.02); TG: *P* = 0.09, b = 0.05, 95% CI (−0.01, 0.11)) after adjustment for age, gender and BMI (Table 5). These results indicated that the reduced insulin sensitivity in rs1051295 genotype TT might at least be associated with the higher TG and more unfavorable W/H ratio.

Table 5 demonstrates that rs1051295 genotype TT exhibited increased fasting insulin level (*P* = 0.004, b = 0.86, 95% CI (0.27, 1.44)) and beta-cell secretory function (*P* = 0.004, b = 0.08, 95% CI (0.03, 0.13)) compared with genotype CC at basal condition. To investigate whether the insulin secretory function of beta-cell in the carrier of genotype TT is superior to genotype CC, we performed OGTT to assess the compensatory secretory function and insulin sensitivity of beta-cells after a glucose challenge. Because of the small size of the groups, and genotype CC and TC demonstrated the similar phenotype in insulin sensitivity in the dominant model at basal glucose, we combined CC and TC as one to compare its insulin sensitivity with genotype TT.

**Figure 1:**
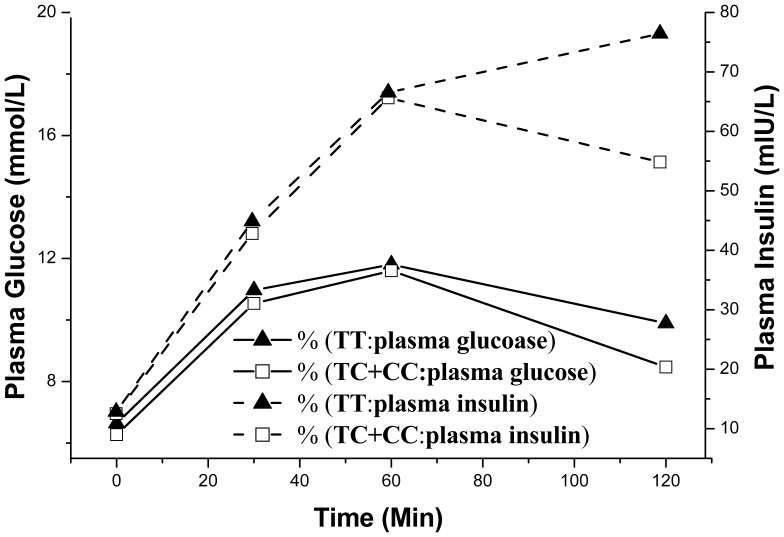
Relationship between plasma insulin and glucose levels during OGTT (0, 30th, 60th and 120th min). n = 23 for rs1051295 genotype TT and 61 for rs1051295 genotypes CC+TC.

OGTT (Table 6) showed there are no significant difference between beta-cell function (ISSI-2) in genotype TT (32.23) and TC+CC (37.54, *P* = 0.13, b = −0.29, 95% CI (−0.67, 0.09)). OGTT also demonstrated genotype TT displayed decreased insulin sensitivity (Matsuda index: 5.05) by 16.3% compared to CC+TC (Matsuda index: 6.03) (*P* = 0.03, b = −1.34, 95% CI (−2.56, −0.12)). This is most dramatically seen at 120min where insulin secretion from genotype TT (76.45 mmol/L) was 28.2% higher than genotype CC+TC (54.84 mmol/L), whereas blood glucose levels at this time point were still 14.4% higher in genotype TT (9.9 mmol/L) than CC+TC (8.5 mmol/L) [Fig. 1]. These results indicate that subjects with rs1051295 genotype CC/TC exhibited greater insulin sensitivity than genotype TT during a glucose load and confirmed the finding at basal condition.

**Table 6:** Comparison of T2D-related traits between rs1051295 genotype CC+TC and genotype TT in a type 2 diabetic-suspected population undergoing an OGTT.

Variable	TT	TC+CC	*P^1^*	*P^2^*	b (95% CIs)
N	23	61			
M/F	9/14	25/36	0.82		
Age	54.70±9.90	51.92±10.64	0.29		
BMI(kg/m^2^)	24.07±3.06	25.01±2.66	0.18		
Glucose0(mmol/L)	6.64±1.82	6.27±1.28	0.30		
Glucose30(mmol/L)	10.97±2.98	10.54±2.55	0.54		
Glucose60(mmol/L)	11.81±3.90	11.60±3.67	0.85		
Glucose120(mmol/L)	9.89±4.44	8.47±3.45	0.13		
Insulin0(mmol/L)*	12.84(9.94,16.06)	12.53(9.83,15.25)	0.61		
Insulin30(mmol/L)*	44.87(33.41,75.59)	42.83(27.25,71.73)	0.36		
Insulin60(mmol/L)*	66.53(49.38,107.76)	65.64(43.42,98.72)	0.74		
Insulin120(mmol/L)*	76.45(33.74,106.15)	54.84(35.32,74.54)	0.10		
ISSI-2*	32.23(26.67,37.71)	37.54(21.45,50.33)	0.30	0.13	−0.29 (−0.67,0.09)
Matsuda index	5.05±1.94	6.03±2.38	0.11	**0.03**	−1.34 (−2.56, −0.12)

M/F: male/female. W/H ratio: waist/hip circumference. b: unstandardized coefficients. CI: confidence interval. *P^1^*: from one-way ANOVA test except for *P* for M/F from χ^2^ test; *P^2^*, b and 95% CIs were from multiple linear regression analysis and adjusted for age, gender, and BMI. *presented as median and quartiles and natural Log-transferred for analysis.

## Discussion

In summary, we unexpectedly found that KCNB1 rs1051295 genotype TT was associated with decreased insulin sensitivity at basal conditions in a general population. Of the three variants in KCNB1 rs1051295, CC (99.9%) and to a less extent of TC (95.2%) exhibited normal insulin sensitivity at basal condition, whereas TT genotype (90.1%), exhibited reduced insulin sensitivity at the fasting state compared to CC (*P* = 0.008, b = −0.09, 95% CI (−0.16, −0.02)) and TC (*P* = 0.096, b = −0.04, 95% CI (−0.09, 0.01)). genotype TT also decreased insulin sensitivity compared with genotype(CC+TC) (allele C)(*P* = 0.05, b = −5.22, 95% CI = (−10.49, −0.00)). This was also confirmed in a fasting glucose impaired population at glucose loading condition. In the OGTT, the TT genotype also exhibited lower insulin sensitivity compared with genotype (TC+CC) (*P* = 0.03, b = −1.34, 95% CI (−2.56, −0.12)). Collectively, these results indicate that KCNB1 rs1051295 TT confers on its carriers the phenotype of decreased insulin sensitivity that is likely to increase the risk of T2D.

Kv2.1, encoded by KCNB1, is the major beta-cell Kv channel in humans [13], [22] and rodents [13], [14] accounting for >70% of outward K^+^ currents. Kv2.1 deletion in mice caused severe perturbation in insulin release and blood glucose [14]. It is reasonable to expect the rs1051295 genotype to be associated with an increased the risk of T2D by impairing islet beta-cell secretory function. We compared the beta-cell function among rs1051295 genotypes at basal condition, whereby HOMA-B% (in vivo assessment of beta-cell function) paradoxically showed a higher value in genotype TT than CC (*P* = 0.004, b = 0.08, 95% CI (0.03, 0.13)).This should not be interpreted to represent a superior beta cell secretory function of the TT genotype, but rather, the higher HOMA-B% in the TT genotype indicates a compensatory action of the pancreatic islet in response to the reduced insulin sensitivity. To confirm this thinking, we compared the beta-cell function under glucose loading condition, and employed the ISSI-2 assessment as a more accurate measurement of beta-cell compensatory insulin secretory function. ISS1-2 assessment showed the KCNB1 rs1051295 CC and TC genotypes was 16.31% higher than TT (Table 6) (*P* = 0.13, b = −0.29, 95% CI (−0.67, 0.09)), suggesting that the TT genotype might have actually reduced insulin secretory capacity, at least with increased glycemic demand during OGTT, and presumably after a meal.

In this study we unexpectedly found that insulin sensitivity was significantly reduced in the KCNB1 rs1051295 genotype TT compared with genotypes CC and TC. Major insulin sensitive tissues are skeletal muscle, adipose tissue and liver. While Kv2.1 is known to be present in skeletal muscle to regulate membrane excitability [23], it is not known to regulate GLUT4 vesicle transport and exocytosis. Recent work demonstrating that Kv2.1 directly interacts with exocytotic SNARE proteins to modulate exocytosis [19], [24], [25] raises the possibility of non-channel exocytotic function(s) of Kv2.1 in skeletal muscle regulating GLUT4 transport or function. Kv2.1 is abundant in neuronal tissue [12] which might influence insulin-sensitive tissues.

We also showed that rs1051295 genotype TT is associated with higher TG levels and affected fat distribution towards an unfavorable waist/hip ratio and abdominal obesity. It is not clear how Kv2.1 could affect adipose tissue metabolism and distribution. We demonstrated the mean HDL-C (TT: 1.65 versus CC: 1.67, *P* = 0.47) was lower and LDL-C (TT: 2.72 versus CC: 2.63, *P* = 0.46) was higher in the population with a TT genotype than those with a CC genotype, which may provide some clues for future investigation on how rs1051295 genotype TT might influence fat metabolism. In support of a role of Kv channels in adipocyte metabolism, Kv channel activity has been recorded in human adipocytes [26], which responded to insulin by increasing channel density [27]. However, the specific Kv isoform or its downstream actions in adipocytes have not been critically assessed, thus requiring much further study. Rs1051295 is located in the 3′-UTR region of KCNB1, and has been found implicated in rheumatoid arthritis [28]. Since this SNP is not located in the coding regions, we speculate that it might not influence channel pore kinetics *per se*, but rather it may influence the post translational processing of the Kv2.1 channel. Further study should be aimed at elucidating at identifying the target tissues that rs1051295 exert its effect on insulin sensitivity.

This work has thus identified KCNB1 rs1051295 genotype TT to be associated with reduced insulin sensitivity and increased plasma TG and W/H ratio, these together likely leading to increase the risk of T2D. Further confirmation will be needed for this study to be performed in a larger population and also to determine if these results could be replicated in other (non-Han) populations. Nonetheless, this initial study opens a new avenue to explore a possible role of Kv2.1 and other Kv channel in influencing fat metabolism and insulin sensitivity.

## Supporting Information

Table S1 **T2D-related quantitative traits in different genotypes of KCNB1 rs1051295 in 226 case-control study.**
(DOC)

Table S2 **T2D-related quantitative traits in different genotypes of KCNB1 rs1051295 in 412 validation study.**
(DOC)
