# Supplementary material for: An Exploratory Study of the Association between KCNB1 rs1051295 and Type 2 Diabetes and Its Related Traits in Chinese Han Population
Source: PLoS One. 2013 Feb 19;8(2):e56365. doi: 10.1371/journal.pone.0056365 (PMC3576392; doi:10.1371/journal.pone.0056365)
Supplement: Table S1 — T2D-related quantitative traits in different genotypes of KCNB1 rs1051295 in 226 case-control study. (DOC) [file pone.0056365.s001.doc]

Table S1. T2D-related quantitative traits in different genotypes of KCNB1 rs1051295 in 226 case-control study

| Variables | Cases | | | |  | Controls | | | |
| --- | --- | --- | --- | --- | --- | --- | --- | --- | --- |
|  | TT | TC | CC | *P* |  | TT | TC | CC | *P* |
| Age | 69.68±11.57 | 66.98±11.49 | 61.85±18.88 | 0.18 |  | 72.91±12.85 | 68.24±16.07 | 69.14±19.65 | 0.21 |
| M/F | 17/23 | 33/35 | 7/8 | 0.83 |  | 22/8 | 35/27 | 13/10 | 0.27 |
| BMI (kg ⁄m^2^) | 26.01±4.18 | 25.11 ±4.31 | 25.31±3.68 | 0.23 |  | 20.76±3.38 | 23.41±3.79 | 21.90±3.32 | 0.06 |
| W/H ratio | 0.90±0.07 | 0.89±0.07 | 0.90±0.05 | 0.81 |  | 0.88±0.06 | 0.88±0.08 | 0.93±0.17 | 0.23 |
| Fasting glucose (mmol/L) | 8.80±3.01 | 9.35±3.03 | 9.12±2.43 | 0.72 |  | 5.03±0.79 | 5.24±0.71 | 4.90±0.65 | 0.30 |
| Triglycerides (mmol/L) | 2.41±2.03 | 2.15±1.64 | 2.47±1.80 | 0.33 |  | 1.24±0.65 | 1.31±0.63 | 1.27±0.57 | 0.50 |
| Systolic Pressure (mmHg) | 130.00±12.35 | 131.79±18.49 | 127.69±7.25 | 0.74 |  | 128.18±20.33 | 120.33±14.41 | 122.43±16.51 | 0.36 |
| Diastolic Pressure (mmHg) | 75.21±9.24 | 76.07±10.21 | 76.54±10.64 | 0.88 |  | 75.36±13.04 | 74.19±12.17 | 71.29±8.28 | 0.87 |

M/F: male/female. W/H ratio: waist/hip circumference ratio. *P*: from one-way ANOVA test except for *P* for M/F from χ^2^ test.
