# Supplementary material for: An Exploratory Study of the Association between KCNB1 rs1051295 and Type 2 Diabetes and Its Related Traits in Chinese Han Population
Source: PLoS One. 2013 Feb 19;8(2):e56365. doi: 10.1371/journal.pone.0056365 (PMC3576392; doi:10.1371/journal.pone.0056365)
Supplement: Table S2 — T2D-related quantitative traits in different genotypes of KCNB1 rs1051295 in 412 validation study. (DOC) [file pone.0056365.s002.doc]

Table S2. T2D-related quantitative traits in different genotypes of KCNB1 rs1051295 in 412 validation study

| Variables | Cases | | | |  | Controls | | | |
| --- | --- | --- | --- | --- | --- | --- | --- | --- | --- |
|  | TT | TC | CC | *P* |  | TT | TC | CC | *P* |
| Age | 68.14±13.42 | 64.29±13.31 | 57.83±16.17 | 0.01 |  | 65.95±14.84 | 62.55±14.35 | 62.52±18.18 | 0.36 |
| M/F | 28/29 | 48/48 | 11/12 | 0.98 |  | 32/23 | 63/59 | 24/24 | 0.65 |
| BMI (kg ⁄m^2^) | 25.95±4.72 | 25.17 ±4.14 | 25.27±3.09 | 0.56 |  | 22.84±4.13 | 24.01±4.16 | 24.00±4.04 | 0.23 |
| W/H ratio | 0.89±0.08 | 0.89±0.07 | 0.89±0.05 | 0.71 |  | 0.89±0.13 | 0.88±0.08 | 0.88±0.07 | 0.97 |
| Fasting glucose (mmol/L) | 8.82±3.31 | 9.41±4.03 | 8.40±2.07 | 0.39 |  | 5.50±0.83 | 5.44±0.70 | 5.59±0.76 | 0.50 |
| Triglycerides (mmol/L) | 2.22±1.84 | 1.92±1.40 | 2.17±1.55 | 0.51 |  | 1.47±0.93 | 1.34±0.63 | 1.37±0.66 | 0.62 |
| Systolic Pressure (mmHg) | 128.75±14.72 | 132.09±16.83 | 125.71±7.46 | 0.16 |  | 127.04±16.11 | 124.09±16.71 | 128.15±17.33 | 0.34 |
| Diastolic Pressure (mmHg) | 75.38±9.13 | 76.17±9.80 | 75.95±9.92 | 0.89 |  | 76.43±11.78 | 76.25±12.14 | 75.00±11.29 | 0.82 |

M/F: male/female. W/H ratio: waist/hip circumference ratio. *P*: from one-way ANOVA test except for *P* for M/F from χ^2^ test.
